# Reconfiguration of Dynamic Functional Connectivity States in Patients With Lifelong Premature Ejaculation

**DOI:** 10.3389/fnins.2021.721236

**Published:** 2021-09-13

**Authors:** Jiaming Lu, Qian Chen, Danyan Li, Wen Zhang, Siyan Xing, Junxia Wang, Xin Zhang, Jiani Liu, Zhao Qing, Yutian Dai, Bing Zhang

**Affiliations:** ^1^Department of Radiology, The Affiliated Drum Tower Hospital of Nanjing University Medical School, Nanjing, China; ^2^Department of Radiology, Nanjing Drum Tower Hospital, Clinical College of Nanjing Medical University, Nanjing, China; ^3^Department of Andrology, The Affiliated Drum Tower Hospital of Nanjing University Medical School, Nanjing, China; ^4^Institute of Brain Science, Nanjing University, Nanjing, China

**Keywords:** premature ejaculation, dynamic functional connectivity, reoccurrence times, frequent transitions, classification

## Abstract

**Purpose:** Neuroimaging has demonstrated altered static functional connectivity in patients with premature ejaculation (PE), while studies examining dynamic changes in spontaneous brain activity in PE patients are still lacking. We aimed to explore the reconfiguration of dynamic functional connectivity (DFC) states in lifelong PE (LPE) patients and to distinguish LPE patients from normal controls (NCs) using a machine learning method based on DFC state features.

**Methods:** Thirty-six LPE patients and 23 NCs were recruited. Resting-state functional magnetic resonance imaging (fMRI) data, the clinical rating scores on the Chinese Index of PE (CIPE), and intravaginal ejaculatory latency time (IELT) were collected from each participant. DFC was calculated by the sliding window approach. Finally, the Lagrangian support vector machine (LSVM) classifier was applied to distinguish LPE patients from NCs using the DFC parameters. Two DFC state metrics (reoccurrence times and transition frequencies) were introduced and we assessed the correlations between DFC state metrics and clinical variables, and the accuracy, sensitivity, and specificity of the LSVM classifier.

**Results:** By k-means clustering, four distinct DFC states were identified. The LPE patients showed an increase in the reoccurrence times for state 3 (*p* < 0.05, Bonferroni corrected) but a decrease for state 1 (*p* < 0.05, Bonferroni corrected) compared to the NCs. Moreover, the LPE patients had significantly less frequent transitions between state 1 and state 4 (*p* < 0.05, uncorrected) while more frequent transitions between state 3 and state 4 (*p* < 0.05, uncorrected) than the NCs. The reoccurrence times and transition frequencies showed significant associations with the CIPE scores and IELTs. The accuracy, sensitivity, and specificity of the LSVM classifier were 90.35, 87.59, and 85.59%, respectively.

**Conclusion:** LPE patients were more inclined to be in DFC states reinforced intra-network and inter-network connection. These features correlated with clinical syndromes and can classify the LPE patients from NCs. Our results of reconfiguration of DFC states may provide novel insights for the understanding of central etiology underlying LPE, indicate neuroimaging biomarkers for the evaluation of clinical severity of LPE.

## Introduction

Premature ejaculation (PE), classified as either lifelong PE (LPE) or acquired PE, is considered one of the most common male sexual dysfunctions (Laumann et al., 1999). According to a large observational study, 25.80% of men complained of PE in China (Gao et al., 2013). Defined as ejaculation within 1 min of vaginal penetration (Serefoglu et al., 2014b), LPE has exerted a significant psychological burden on men, their partners, and the male/partner relationship (Rowland et al., 2007).

Despite the high prevalence and negative impacts of PE, the mechanisms underlying the pathophysiology of PE have not yet been entirely elucidated. It is well documented that a complex interplay among 5-hydroxytryptamine_2C_ (5-HT_2C_) receptor hyposensitivity and/or 5-hydroxytryptamine_1A_ (5-HT_1A_) receptor hypersensitivity, genetic components, hormonal factors, and urological conditions contributes to the disruption of highly coordinated ejaculation processes and the lower ejaculatory threshold of PE (Saitz and Serefoglu, 2015).

Magnetic resonance imaging (MRI) has been proven to be an objective and effective approach to investigate the structural and functional neural basis of PE. Previous neuroimaging studies have reported several factors in PE patients: marked cortical thickening over areas in the frontal, parietal, and occipital lobes and the cingulate cortex (Guo et al., 2018); significantly larger mean volume of the caudate nucleus (Atalay et al., 2019); altered DTI structural connectivity of the fronto-cingulate-parietal control network (Gao et al., 2018; Chen et al., 2021); lower activation in the left inferior frontal gyrus and left insula, and higher activation in the right middle temporal gyrus when exposed to erotic picture stimuli (Zhang et al., 2017); widespread increases in fractional anisotropy and axial diffusivity values in the right posterior thalamic radiation, posterior corona radiata and bilateral posterior limb of the internal capsule (Gao et al., 2018); and decreased local efficacy in the left amygdala, right pallidum, and thalamus, as well as decreased global efficacy in the left amygdala and right rolandic operculum (Chen et al., 2020a). In addition, studies focused on functional connectivity (FC) in PE patients have observed decreased short-range functional connectivity density (FCD) in the bilateral middle temporal gyrus, left orbitofrontal cortex, and nucleus accumbens and increased long-range FCD in the left insula, Heschl’s gyrus, and putamen (Lu et al., 2018); decreased hypothalamus-seeded FC in the left orbitofrontal cortex, bilateral insula, and superior temporal cortex (Gao et al., 2020b); and weaker left inferior frontal gyrus (IFG)-seeded FC in the left dentate nucleus and right frontal pole (Yang et al., 2018). The aforementioned findings suggested altered structural substrates and functional activities of the cortical-subcortical pathways involved in ejaculation processing in PE patients. These investigations may benefit the understanding of the central pathological mechanism underlying PE; however, the results are rarely consistent. This may be partly attributable to the factor that ejaculation is a complex process involving the functional segregation and integration of widespread brain networks (Giuliano, 2011; Chen et al., 2020c). Thus, it may be more appropriate to explore the neural basis underlying PE from the perspective of functionally coordinated brain networks.

Currently, the assessment of PE mainly depends on validated questionnaires such as the Chinese Index of Sexual Function for Premature Ejaculation (CIPE)-5 (Yuan et al., 2004), or patient-reported intravaginal ejaculatory latency time (IELT) (El-Hamd et al., 2019), which influenced by manual involvement and potential bias. A study combining static FC (SFC) and machine learning reported an accuracy of 0.895 ± 0.12 for distinguishing LPE patients from normal controls (NCs) and concluded that classification features of SFC might be helpful to discriminate LPE patients and provide abnormal central functional targets in LPE etiology (Xu et al., 2019). However, fMRI researchers have been motivated by the perspective that the brain is inherently a dynamic system (Hutchison et al., 2013; Allen et al., 2014). Therefore, previous neuroimaging studies based on SFC may be limited in their ability to reveal the dynamic changes in spontaneous brain activity in PE patients. Instead, dynamic functional connectivity (DFC) can exhibit highly structured spatiotemporal patterns in which a set of metastable FC patterns, known as DFC states, reliably recur across time and subjects (Allen et al., 2014; Li et al., 2017). Altogether, DFC has been suggested to be a more informative representation of functional brain networks than SFC (Peraza et al., 2015; Schumacher et al., 2019). DFC states have been suggested to be associated with high levels of thought or consciousness (Hutchison et al., 2013), flexible behaviour (Tognoli and Kelso, 2014), neuropsychiatric disorders (Damaraju et al., 2014), and development (Hutchison and Morton, 2015). Two commonly described measures in DFC studies were reoccurrence times of distinct states and the number of transitions between states, which have been suggested significantly associated with neuropsychological, behavioral, and clinical variables (Allen et al., 2014; Liu et al., 2017; Fiorenzato et al., 2019). The latter could reflect neural metastability at another level, that is, enabling multiple brain regions to flexibly engage and disengage in coordination without being locked into fixed interaction patterns (Li et al., 2017). Since ejaculation is considered a coordinated activity associates with both neuropsychological and behavioral pathways within the neural circuits (Chen et al., 2020b), we speculated that such processes may require the flexible transitions between distinct DFC states. Instead of the task fMRI data, we chose the resting state fMRI data, our hypotheses were as follows: (1) certain DFC state patterns and alterations in DFC state metrics (reoccurrence times and transition frequencies) would be observed in LPE patients; (2) the altered DFC state metrics would be associated with clinical rating scale scores; and (3) the classifier based on DFC state features could discriminate LPE patients from NCs at a high accuracy. In the current study, we focused on exploring the reconfiguration of DFC states in LPE patients and tried to distinguish LPE patients from NCs using the Lagrangian support vector machine (LSVM) classifier, which based on the linearly convergent learning algorithm with high accuracy, stable performance, and fast learning speed (training time) (Mangasarian and Musicant, 2001; Czabanski et al., 2020), based on the feature selection of DFC parametric.

## Materials and Methods

### Subjects

From 2013 to 2016 in Nanjing Drum Tower Hospital, 36 right-handed patients with LPE were recruited, and 23 NCs were enrolled in this study. All the PE patients were recruited from andrology department and the NC participants were recruited from the community. Some data from the datasets have been reported in our previous work (Zhang et al., 2017; Lu et al., 2018). The ISSM guidelines were applied for the diagnosis of LPE patients (Serefoglu et al., 2014a). More specifically, LPE patients were characterized by (a) ejaculation that always or nearly always occurs prior to or within about 1 min of vaginal penetration; (b) the inability to delay ejaculation on all or nearly all vaginal penetrations; and (c) negative personal consequences such as distress, bother, frustration, and/or the avoidance of sexual intimacy. Twenty three subjects were recruited as NCs, with self-reported IELTs of more than three min. All the subjects were in a stable relationship with the same, non-pregnant, sexually active partner for at least 1 year. All participants completed the International Index of Erectile Function (IIEF)-5 questionnaire (Rhoden et al., 2002) to assess the erectile function, the CIPE-5 questionnaire (Yuan et al., 2004) (including 5 questions focused on ejaculatory latency, sexual satisfaction, difficulty in delaying ejaculation, and depression, with each question responding to on a 5 point Likert-type scale). Participants with IIEF-5 score < 21, reduced sexual desire, inhibited male orgasm, mental disorders, systemic or neurological problems, physical illnesses which affect ejaculatory function, abuse of alcohol, and any medical treatment for PE in the past 6 months were excluded.

### Image Acquisition

The imaging data were acquired with a 3T Achieva TX MRI system (Achieva 3.0 T TX, Philips Medical Systems, Eindhoven, Netherlands), and the detailed acquisition parameters of rs-fMRI data were set as follows: field of view (FOV) = 192 × 192 mm^2^; section thickness = 4 mm with no section gap; matrix = 64 × 64; repetition time (TR) = 2,000 ms; echo time (TE) = 30 ms; and flip angle = 90°. There were 12 dummy scans, then 230 volumes were acquired followed. During rs-fMRI scanning, each subject was asked to lie quietly with their eyes closed. In addition, we acquired high-resolution 3D T_1_-weighted brain structural images with the following parameters: TR = 7,600 ms; TE = 3,400 ms; flip angle = 8°; FOV = 256 × 256 × 192 mm^3^ and slice thickness = 1 mm.

### Image Preprocessing

Rs-fMRI data preprocessing was performed by the Data Processing and Analysis for Brain Imaging (DPABI, vision 5.0)^1^ (Yan et al., 2016). Slice timing, realignment, nuisance regression (white matter and cerebrospinal fluid signals and rigid-body 6 head motion parameters), and spatial normalization to the standard Montreal Neurological Institute (MNI) echo-planar imaging (EPI) template with a resampled voxel size of 3 × 3 × 3 mm^3^ were conducted for all 230 time points. We did not perform global signal regression to avoid introducing distortions in the time series data (Murphy et al., 2009; Anderson et al., 2011). Then, the data were smoothed with an 8-mm full width at half maximum Gaussian kernel, detrended to remove the linear trend of time courses, and band-pass filtered (0.01–0.10 Hz voxel by voxel) to reduce the effects of low-frequency drift and high-frequency respiratory and cardiac noise. No participant was excluded with head motion ≥ 3 mm/3°. Two-sample *t*-tests indicated no significant differences in the mean framewise displacement (Jenkinson) (Jenkinson et al., 2002) between the LPE and NC groups (0.090 ± 0.055 mm versus 0.087 ± 0.054 mm, *p* = 0.856).

### Dynamic Functional Connectivity Network

**FIGURE 1:**
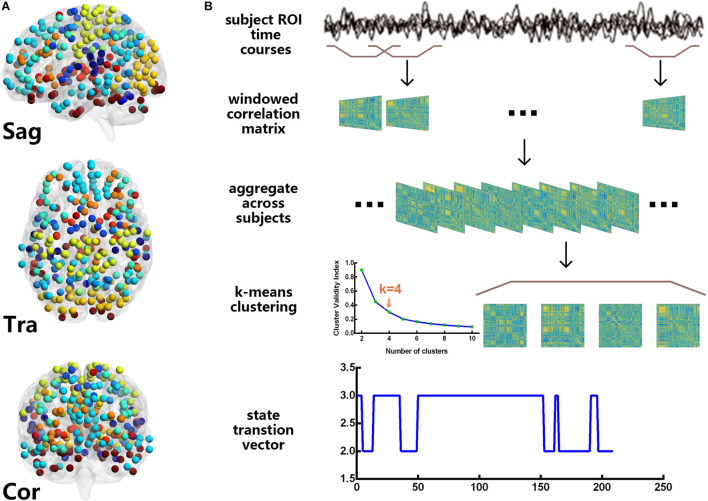
Power 264 atlas **(A)** and the flowchart of the dynamic functional connectivity analysis **(B)**. For these resting-state functional magnetic resonance imaging data, we used the sliding window approach to obtain 209 time windows and clustered the windows for all participants by the k-means algorithm. The cluster centroids and cluster membership assignments that were obtained for all the windows represented the state transition vector.

Figure 1 shows the flowchart of the DFC network analysis in this study. The main manipulations can be described as follows:

### Sliding Window Approach

The Power 264 atlas (Power et al., 2011) was used to calculate the DFC, which contains thirteen brain subnetworks, including the auditory (AUD), cerebellar (CB), cingulo-opercular task control (CON), dorsal attention (DAN), default mode (DMN), fronto-parietal task control (FPN), memory retrieval (Mem), sensory/somatomotor hand (Motor), visual (Vis), salience (SN), subcortical (Sub), ventral attention (VAN), and other networks. Here, we used the Power 264 atlas as a template, and the sliding window approach (Barttfeld et al., 2015; Karahanoğlu and Van De Ville, 2015) was applied to estimate the DFC network for each subject, resulting in (264 × (264–1))/2 = 34,716 features. We created tapered windows by convolving a rectangle (length = 22 TRs) with a Gaussian of σ = 3 TRs because previous studies have shown that a window length of 22 TRs provided a good trade-off between the quality of the functional network connectivity estimate and the temporal resolution (Allen et al., 2014). The onset of each window progressively slid in steps of 1 TR from that of the previous one, resulting in 209 windows. In the calculations, we also imposed an L1 penalty on the precision matrix (inverse of the correlation matrix) to enhance sparsity (Smith et al., 2009).

### K-Means Clustering

Because the DFC patterns reoccur within the subjects across time and the subjects, we applied the k-means algorithm to divide the DFC windows into separate clusters (Allen et al., 2014; Barttfeld et al., 2015). Before clustering, we estimated the variability in the DFC across all pairs at each window, and subject exemplars were chosen as those windows with local maxima in FC variance. Then, we performed a k-means analysis on the set of all the subject exemplars with random initialization of the centroid positions. For this analysis, *k* = 4 was determined using the elbow criterion, defined as the ratio of within cluster to between cluster distances (Damaraju et al., 2014). The correlation distance function was chosen because it is more sensitive to the DFC pattern, regardless of magnitude (Allen et al., 2014). These resulting centroids were then used as starting points to cluster the DFC windows for all the subjects.

In this study, the properties of the DFC of each subject were depicted by two metrics, the reoccurrence times for all DFC states and the DFC transition frequencies between all the pairs of the DFC states. For each subject, the reoccurrence times for each DFC state were defined as the total number of DFC windows assigned to that state, and the DFC transition frequency counts how many times the DFC windows altered their allegiance between a pair of two successive states (e.g., the transition frequency between DFC state 1 and state 2 counted if the DFC windows altered their allegiance from state 1 to state 2 or from state 2 to state 1).

### Feature Selection Lagrangian Support Vector Machine-Based Classification

An LSVM method was applied to classify LPE patients from NCs using the number of states, reoccurrence times and the transition frequencies as features. The 10-folds cross validation was adopted to evaluate the classification performance, which provides a good estimation for the generalizability of the classifiers, particularly when the sample size is small (Pereira et al., 2016). The selection of the discriminative features and elimination of the non-informative features has been widely employed to boost classification performance (Dosenbach et al., 2010; Dai et al., 2012). This study applied a nested 10-folds cross validation using the outer loop to estimate classification accuracy and the inner loop to determine the optimal feature selection (Whelan et al., 2014; Hahn et al., 2015). The details of this framework were described in a previous study (Vapnik, 2013), including feature selection, LSVM implementation, and evaluation of classification performance, and codes can be found at https://github.com/ZaixuCui/HBM_Dyslexia_Classification.

### Statistical Analysis

Age, body mass index (BMI), and clinical rating scale scores were compared using two-sample *t*-tests, while education level and marital status were calculated by chi-square tests. Group differences in the reoccurrence times for each DFC state and the transition frequencies between each pair of DFC states were also estimated using two-sample *t*-tests. We further calculated the Pearson’s correlations between DFC state metrics and clinical variables of IELTs and CIPE scores. The associations between DFC metrics and mean framewise displacement (Jenkinson) were also assessed. Statistical analyses were performed with SPSS version 21.0 (IBM Corp., Armonk, NY, United States), and *p* < 0.05 was set as a statistically significant value. Mann-Whitney *U*-test was used for the non-parametric tests. Bonferroni corrections were applied for multiple comparisons.

## Results

### Demographic and Clinical Data

Demographic, psychiatric, and behavioral features for the LPE patients and NCs are listed in Table 1. As shown, the two groups did not significantly differ in age, marital status, BMI, or education level. The IIEF-5 scores also showed no significant difference between the groups, indicating the preserved erectile function in LPE patients. In contrast, the LPE patients showed significantly lower CIPE scores (*p* < 0.01) and shorter IELTs (*p* < 0.01) than NCs. Furthermore, the scores of each CIPE question were also significantly lower in the LPE patients than in the NCs.

**TABLE 1:** Demographic and clinical data of LPE patients and NCs.

	LPE (*n* = 36)	NC (*n* = 23)	Statistics	*p*
**Age (years)**				
Mean ± SD	27.61 ± 4.48	26.39 ± 4.11	1.053	0.297
**Marital status (%)**				
Single/Married	52.78/47.22	60.87/39.13	0.373	0.541
**BMI (kg⋅m^–2^)**				
Mean ± SD	23.81 ± 3.17	23.47 ± 2.95	0.413	0.681
Education level (%)			0.094	0.954^a^
Elementary	11.11	8.70		
High school	38.89	39.13		
University	50.00	52.17		
**IIEF-5 score**				
Mean ± SD	23.78 ± 1.31	24.17 ± 0.65	−1.344	0.184
**CIPE**				
Total	9 (7, 10)	22 (20, 24)	828.00	<0.001
Q1	2 (1, 2)	5 (5, 5)	828.00	<0.001
Q2	1 (1, 1)	4 (3, 5)	828.00	<0.001
Q3	1 (1, 1)	4 (4, 5)	828.00	<0.001
Q4	1 (1, 1)	4 (4, 5)	828.00	<0.001
Q5	4 (2.25, 4)	5 (4, 5)	600.00	0.001
**IELT (minutes)**				
Mean ± SD	0.80 ± 0.40	9.96 ± 4.47	−12.269	<0.001

*The data from questionnaires were presented in terms of the mean scores (Mean) and standard deviations (SD) in the lifelong premature ejaculation (LPE) and normal control (NC) groups. Mann- Whitney U-test was used for IIEF score comparison, data were presented in terms of the media (25th quartile, 75th quartile). IIEF-5, International Index of Erectile Function-5; CIP, Chinese Index of Premature Ejaculation; IELT: intravaginal ejaculatory latency time; BMI, body mass index.*

*^a^According to the years of education each subject accepted, they were classified as elementary (0–9 years), high school (9–12 years), and university (>12 years) education level.*

### Connectivity Patterns of the Dynamic Functional Connectivity States

**FIGURE 2:**
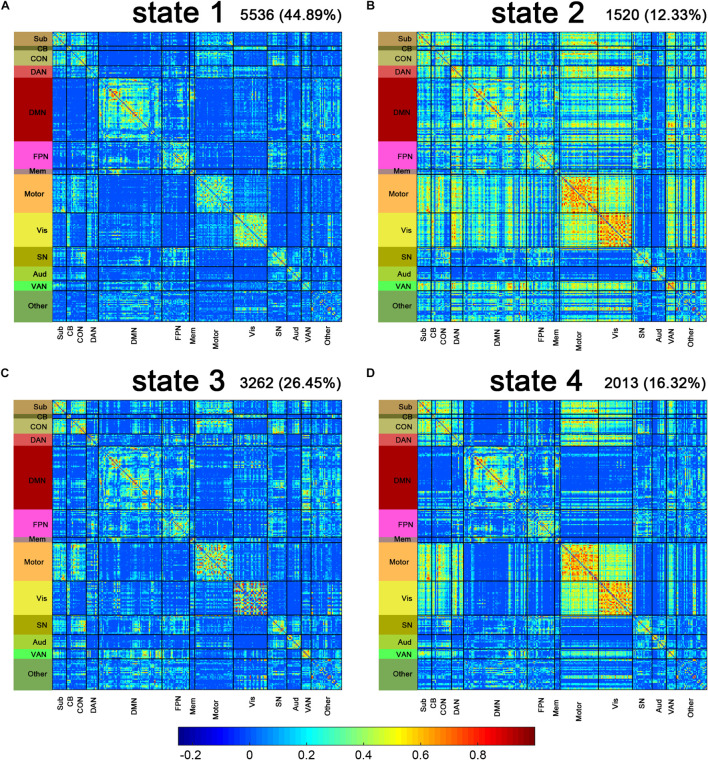
Connectivity patterns of the dynamic functional connectivity (DFC) states. The centroids of the DFC states are shown above **(A–D)**. The total number and percentage of occurrences of each state are listed above each centroid. Sub, subcortical; CB, cerebellar; CON, cingulo-opercular task control; DAN, dorsal attention; DMN, default mode; FPN, fronto-parietal task control; Mem, memory retrieval; Motor, sensory/somatomotor hand; Vis, visual; SN, salience; Aud, auditory; VAN, ventral attention.

Figure 2 shows the centroids of the four DFC states. Briefly, state 1 showed a predominance of moderate correlations within most of the networks, especially the fronto-parietal task control network (CON), default mode network (DMN), somatomotor network (Motor), and Visual (vis) networks, while weak connectivity between networks (Figure 2A). State 2 represented a highly connected state. The dorsal attention network (DAN), Motor, Vis, and ventral attention network (VAN) networks showed highly inter-network connection, while the salience network (SN) and auditory network (AUD) networks showed sparse inter-network connections with other networks (Figure 2B). The connectivity pattern in state 3 resembled that in state 1 but showed slightly increased strength within the subcortical network (Sub), CON, Motor, and Vis networks, as well as increased strength between the Sub, CON, and Motor networks (Figure 2C). In state 4, the Sub, CON, Motor, and Vis networks displayed both high inter-network and intra-network connectivity, while other networks showed sparse inter-network connections (Figure 2D). The number and proportion of different states assigned to the LPE and NC groups are shown in Table 2.

**TABLE 2:** The number of windows in each state in the LPE and NC groups.

	State 1	State 2	State 3	State 4	Sum
LPE	2,868 (38.12%)	972 (12.92%)	2,286 (30.38%)	1,398 (18.58%)	7,524
NC	2,668 (55.50%)	548 (11.40%)	976 (20.30%)	615 (12.80%)	4,807
Sum	5,536	1,520	3,262	2,013	12,331

*LPE, lifelong premature ejaculation, n = 36; NC, normal control, n = 23.*

### Group Differences in Dynamic Functional Connectivity State Metrics and the Correlations With Clinical Rating Scale Scores

**FIGURE 3:**
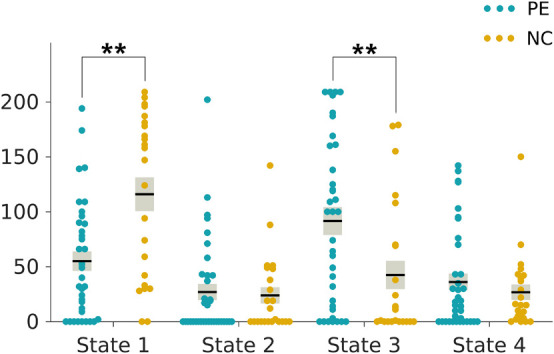
Group differences in reoccurrence times of each dynamic functional connectivity state. The reoccurrence times of each individual in the lifelong premature ejaculation (LPE) and normal control (NC) groups are presented in blue and khaki, respectively. For each group, the black line indicates the mean reoccurrence times in that group, and the light gray rectangle covers the data within one standard error above and below the mean. The pairs of groups with asterisks indicate significant differences between them: **p < 0.05, Bonferroni corrected.

**FIGURE 4:**
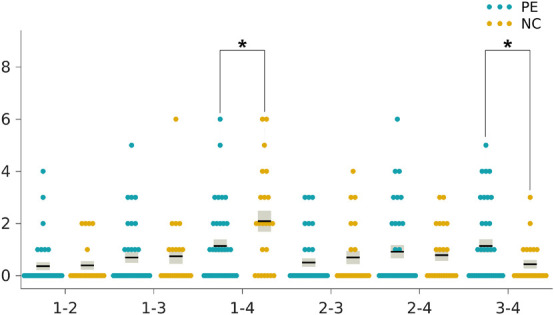
Group differences in the transition frequencies between the dynamic functional connectivity states. The ticks on the horizontal axis indicate the state transition pairs, e.g., “1–2” refers to the transitions between state 1 and state 2. The state transition times of each individual in the lifelong premature ejaculation (LPE) and normal control (NC) groups are presented in blue and khaki, respectively. For each group, the black line indicates the mean state transition times in that group, and the light gray rectangle covers the data within one standard error above and below the mean. The pairs of groups with asterisks indicate significant differences between them: *p < 0.05, uncorrected.

The two-sample *t*-tests indicated an increase in the reoccurrence times for state 3 (*p* < 0.05, Bonferroni corrected) but a decrease for state 1 (*p* < 0.05, Bonferroni corrected) in the LPE patients compared to the NCs (Figure 3). In addition, the LPE group showed significantly less frequent transitions between state 1 and state 4 (*p* < 0.05, uncorrected), while more frequent transitions between state 3 and state 4 (*p* < 0.05, uncorrected) (Figure 4).

As shown in Table 3, the reoccurrence times for state 1 were significantly positively correlated with both CIPE scores and IELTs (*r* = 0.437, *p* = 0.001; *r* = 0.452, *p* < 0.001, respectively, Table 3) after Bonferroni corrections with adjusted α of 0.0025. While the reoccurrence times for state 3 were negatively correlated with CIPE scores and IELTs (*r* = −0.323, *p* = 0.013; *r* = −0.342, *p* = 0.008, respectively) under uncorrected criteria. Furthermore, the number of transitions between state 3 and state 4 showed negative correlations with CIPE scores (*r* = −0.272, *p* = 0.037). No significant associations between DFC metrics and mean framewise displacement (Jenkinson) were observed.

**TABLE 3:** Correlations between dynamic functional connectivity metrics and clinical variables.

	CIPE		IELT	
	*r*	*p*	*r*	*p*
**Reoccurrence times**				
State 1	0.437	0.001**	0.452	< 0.001**
State 2	–0.037	0.779	0.008	0.953
State 3	–0.323	0.013*	–0.342	0.008*
State 4	–0.110	0.407	–0.146	0.271
**Transitions**				
State 1–state 2	0.054	0.684	0.091	0.493
State 1–state 3	–0.052	0.695	–0.020	0.882
State 1–state 4	0.223	0.090	0.230	0.080
State 2–state 3	0.052	0.698	–0.008	0.954
State 2–state 4	0.061	0.647	0.074	0.576
State 3–state 4	–0.272	0.037*	–0.248	0.059

CIP, Chinese Index of Premature Ejaculation; IELT, intravaginal ejaculatory latency time. **p < 0.0025 (Bonferroni adjusted α); *p < 0.05, uncorrected.

### Classification Evaluation

**FIGURE 5:**
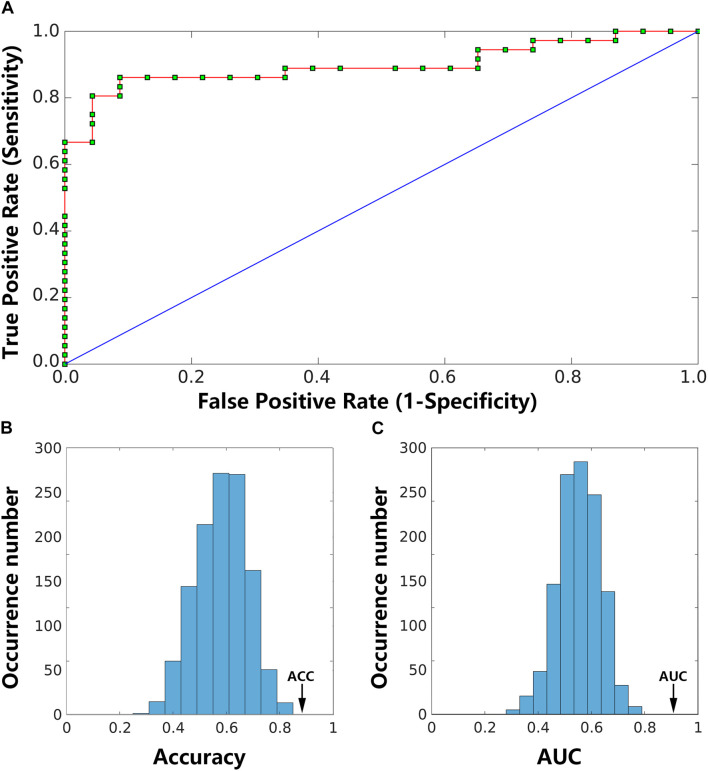
Classification results. The receiver operating characteristic (ROC) curve for the classifier using dynamic functional connectivity (DFC) state features achieved an area under the curve (AUC) of 0.91 **(A)**. Two histograms below are the permutation distribution of the accuracy (ACC; **(B)** and AUC **(C)** for the feature-based classifier. The values obtained using the real labels are indicated by the arrows.

The LSVM classifier accurately discriminated LPE patients from NCs using the number of states, reoccurrence times and the transition frequencies as features. Specifically, the accuracy, sensitivity, and specificity were 90.35, 88.21, and 85.59%, respectively. The permutation test revealed a level of *p* < 0.001 for accuracy (Figure 5B), suggesting significantly higher prediction accuracy than chance. The classification results are shown as a receiver operator characteristic (ROC) curve using each subject’s classification score as a threshold in Figure 5A. The area under the curve (AUC) was 0.91, which was also significantly higher than chance (*p* < 0.001), indicating excellent discriminative power (Figure 5C).

## Discussion

To the best of our knowledge, this is the first study combining DFC analysis and a machine learning classification approach to investigate DFC characteristics in LPE patients and constructed an optimal classifier to distinguish LPE patients from NCs. Our findings suggested that (1) LPE patients were more inclined to be in the DFC state with reinforced intra-network connectivity in the Sub, CON, Motor, and Vis networks, as well as strong inter-network connections among the Sub, CON, and Motor networks compared to the NCs; (2) the DFC state measures of reoccurrence times and transition frequency could reflect the severity of clinical symptoms; and (3) the LSVM classifier based on DFC state features could discriminate LPE patients from NCs at a high accuracy of 0.85. This study may provide novel insights into the dynamic organization principles of brain networks underlying LPE and pave the way for the accurate and automated diagnosis of LPE. This study may help the clinical docker have a better neuroimage mechanism understanding of the LPE disease.

In the present study, the k-means algorithm separated the DFC windows into four distinct states across the entire group, with each state showing a unique pattern as described above. Consistent with previous studies (Kim et al., 2017; Viviano et al., 2017; Fiorenzato et al., 2019; Schumacher et al., 2019; Gu et al., 2020), the most frequently occurred state was a relatively sparse connectivity pattern among the four states, that is state 1 in the present study, characterized by moderate correlations within most of the networks, while the absence of strong correlations between networks, accounting for 44.89% of the total DFC windows. State 1 showed a modularized profile dominated by moderate intra-network connections and may represent the baseline connectivity pattern that participant spent most of their time in, while other states with strong connections may reflect the neuropsychological processes (Viviano et al., 2017; Gu et al., 2020), such as state 2 and state 4 in the present study. State 3, accounted for 30.38% of the DFC states in LPE patients, while 20.30% in NCs, which may represent the LPE-related state.

From the perspective of the two metrics of DFC states, we found that the LPE patients showed significantly fewer reoccurrence times for state 1 while more reoccurrence times for state 3 compared to the NCs. The connectivity pattern in state 3 was similar to the baseline state but showed slightly increased strength within the Sub, CON, Motor, and Vis networks, as well as more connections between the Sub, CON, and Motor networks. The Sub network was believed to function in the dopaminergic pathways and the brain’s reward circuitry to regulate emotional and cognitive behavior (Holstege et al., 2003; Lu et al., 2018; Atalay et al., 2019). Increased dopamine neurotransmission in the subcortical regions might reduce the threshold of ejaculation in PE patients (Chen et al., 2020b). We speculated that increased strength within Sub network implied excessive activity of the dopamine system. Dopamine-related neural activation facilitated the occurrence of ejaculation (Saitz and Serefoglu, 2015), which may be one of the pathological basis underlying LPE. The Vis network is involved in the processing of visual input and was reported to be associated with visual stimuli that induced sexual arousal (Walter et al., 2008). Previous studies have shown increased activation in the visual reward networks underlying the higher sensitivity to explicit visual erotic stimuli in men than women (Lee et al., 2015). We hypothesized that the strength of Vis network connectivity could be related to the sensitivity changes to visual stimulation in PE patients, which may contribute to uncontrollable rapid ejaculation. The increased intra-network connectivity of Motor network may be related to LPE patients are more actively preparing for income stimuli in the resting state. Meanwhile, ejaculation is a multidimensional experience controlled by brain regions involved in the inhibitory processing of sexual stimuli (Zhang et al., 2017). The increased central neural circuitry in the CON network observed in the LPE patients may enhance the inhibitory control over ejaculation, which may serve in a compensatory way for the high sensitivity to sexual stimuli. In addition, the connections between the Sub, CON, and Motor networks were enhanced in LPE patients, which may indicate increased communication and interaction between the excitatory and inhibitory pathways. These findings indicated that the pathological and compensatory mechanisms coexist in patients with LPE, resulting in LPE patients more inclined to be in states with reinforced intra-network and inter-network connectivity in the Sub, CON, and Motor networks.

In addition, the LPE patients showed a significantly less transition frequency between state 1 and state 4, while more transition frequency between state 3 and state 4. We speculated that state 4, with both high inter-network and intra-network connectivity in Sub, CON, DAN, Motor, and Vis networks, may represent the complex neural circuits that partly accountable for ejaculation. The fewer transitions between state 1 and state 4 in LPE patients may indicate the inability to flexibly switch between the baseline state and the ejaculation state to transfer information in a more energy-saving way (Gu et al., 2020). Instead, the LPE patients showed more switches between state 3 and state 4, suggesting the tendency of more transitions between the LPE-related state and the ejaculation state, as well as the high energy-consuming information processing procedure. Notably, the differences in transitions between states did not survive Bonferroni corrections and future research will be needed to confirm these results.

The correlation analyses between DFC state metrics and clinical variables of CIPE scores and IELTs showed that more reoccurrence times for state 1 were associated with higher CIPE scores and longer IELTs. Instead, more reoccurrence times for state 3 were associated with more serious clinical evaluation of LPE, demonstrated by lower scores of CIPE and shorter IELTs. Besides, the number of transitions between state 3 and state 4 was negatively correlated with CIPE scores. These findings were supportive of the suggestion that alterations in DFC state parameters may be cognitively or behaviorally relevant (Liu et al., 2017; Fiorenzato et al., 2019; Gu et al., 2020). We speculated that the two DFC state metrics introduced in the present study could also reflect the clinical severity of LPE from the perspective of objective neuroimaging markers. More specifically, state 1 seemed to represent the central mechanisms of normal ejaculation, while state 3, a core and specific state for LPE, seemed to demonstrate the connectivity pattern of uncontrollable rapid ejaculation. Taken together, these findings confirmed the reconfiguration of rs-fMRI networks and underlined the importance of DFC temporal properties in the evaluation of clinical severity in LPE patients.

Currently, the diagnosis of PE mainly relies on behavioral assessment of IELT, which is typically time-consuming and highly depends on the accuracy of the time recorded by the patient. In contrast, the automated neuroimaging-based machine learning classification method could avoid manual involvement and potential bias and provide valuable evidence of the neural basis underlying LPE. The neuroimaging-based machine learning classification method has previously been applied to a broad range of studies (Cui et al., 2016; Plaschke et al., 2017; Spera et al., 2019), but studies on DFC-derived features have rarely been reported. Abrol et al. (2019) introduced a multimodal (structural MRI and DFC) data fusion framework to predict Alzheimer’s disease progression and found a significant improvement in performance over unimodal prediction analyses. Sakoglu et al. (2019) reported a higher accuracy of DFC-based classification (0.95) than that based on SFC (0.81) for the identification of cocaine dependence, suggesting the diagnostic value of DFC metrics. In the present study, we used the DFC state of each window as features to construct an optimal classifier to distinguish the LPE patients from the NCs. The classifier ended up with a relatively high classification accuracy of 0.85. Therefore, the LSVM classifier based on DFC state features may provide a tool help clinical doctor distinguish LPE patient from NCs with an acceptable accuracy. Despite the advantages of the neuroimaging-based machine learning classification method mentioned above, it cannot be ignored that cranial MRI canning is much more expensive than the self-recorded IELT. The idea that introducing the LSVM classifier to clinical practice is promising but still has lots of challenges.

Several limitations should be considered in the present study. First, the DFC analysis was conducted in a small cohort; thus, the generalization of our findings warrants further validation by studies with large cohorts. Second, this study focused on LPE patients, so whether the current results are applicable to other types of PE is unknown. Caution should be exercised when extrapolating our findings across other types of PE. Third, the time points of rs-fMRI across different research centers may be inconsistent, thus the diagnostic value of DFC measures remains to be further validated by multicenter clinical studies. Notably, the number of state reoccurrence times and transitions between states contained many zero values, which may have an impact on the correlations between DFC temporal properties and clinical measures. Further, the alternations in DFC properties in LPE patients compared to NCs may not totally attributable to the neural basis of PE, since accompanying suffering, frustration, dissatisfaction, and other psychological states may also influence the results (Gao et al., 2020a). The findings observed in the present study were exploratory results that need further validation. Finally, other modality features should also be included to make the classification more comprehensive. Multimodal imaging data should be applied in classification studies of PE in the future.

## Conclusion

To conclude, this is the first study attempting to investigate DFC temporal properties, with a focus on the reoccurrence times and transition frequency, and diagnostic value of a machine learning method based on DFC state features in LPE patients. Importantly, LPE patients were more inclined to be in the state characterized by slightly increased intra-network connectivity in the Sub, CON, Motor, and Vis networks and increased inter-network connectivity among the Sub, CON, and Motor networks, which may provide novel insights for the understanding of central neural basis underlying LPE. The associations between DFC metrics and clinical variables may suggest neuroimaging biomarkers for the evaluation of clinical severity of LPE. Furthermore, the LSVM classifier based on DFC state features achieved a high accuracy of 0.85, which may pave the way for the accurate and automated identification of LPE patients.

## Data Availability Statement

The raw data supporting the conclusions of this article will be made available by the authors, without undue reservation.

## Ethics Statement

The studies involving human participants were reviewed and approved by the Human Participants Ethics Committee of the Nanjing Drum Tower Hospital. The patients/participants provided their written informed consent to participate in this study.

## Author Contributions

JLu was responsible for the conception and design of the present study, execution of the experimental work, and wrote the first draft of the manuscript. QC and DL organized the research project and reviewed and critiqued the manuscript. WZ, SX, and JW executed the experimental work. XZ, YD, ZQ, and BZ reviewed and critiqued the statistical analysis. BZ guided the design of the study protocol, and reviewed and critiqued the manuscript. All authors contributed to the article and approved the submitted version.

## Conflict of Interest

The authors declare that the research was conducted in the absence of any commercial or financial relationships that could be construed as a potential conflict of interest.

## Publisher’s Note

All claims expressed in this article are solely those of the authors and do not necessarily represent those of their affiliated organizations, or those of the publisher, the editors and the reviewers. Any product that may be evaluated in this article, or claim that may be made by its manufacturer, is not guaranteed or endorsed by the publisher.

## Footnotes

1
http://www.restfmri.net
